# Peptide-Based Inhibitors of ADAM and ADAMTS Metalloproteinases

**DOI:** 10.3389/fmolb.2021.703715

**Published:** 2021-07-21

**Authors:** Stefano Pluda, Ylenia Mazzocato, Alessandro Angelini

**Affiliations:** ^1^ Department of Molecular Sciences and Nanosystems, Ca’ Foscari University of Venice, Venice, Italy; ^2^ Fidia Farmaceutici S.p.A., Abano Terme, Italy; ^3^ European Centre for Living Technology (ECLT), Venice, Italy

**Keywords:** A-disintegrin and metalloproteinase (ADAM), A-disintegrin and metalloproteinase with thrombospondin motifs (ADAMTS), metalloproteinase, peptide inhibitors, linear peptides, cyclic peptides, macrocycles

## Abstract

ADAM and ADAMTS are two large metalloproteinase families involved in numerous physiological processes, such as shedding of cell-surface protein ectodomains and extra-cellular matrix remodelling. Aberrant expression or dysregulation of ADAMs and ADAMTSs activity has been linked to several pathologies including cancer, inflammatory, neurodegenerative and cardiovascular diseases. Inhibition of ADAM and ADAMTS metalloproteinases have been attempted using various small molecules and protein-based therapeutics, each with their advantages and disadvantages. While most of these molecular formats have already been described in detail elsewhere, this mini review focuses solely on peptide-based inhibitors, an emerging class of therapeutic molecules recently applied against some ADAM and ADAMTS members. We describe both linear and cyclic peptide-based inhibitors which have been developed using different approaches ranging from traditional medicinal chemistry and rational design strategies to novel combinatorial peptide-display technologies.

## Introduction

The “A-disintegrin and metalloproteinase” (ADAM) and “A-disintegrin and metalloproteinase with thrombospondin motifs” (ADAMTS) are closely related matrix zinc-dependent metalloproteinases that belong to the adamlysin protein family (Takeda, 2016). Most ADAM metalloproteinases are membrane-anchored enzymes while the ADAMTS family comprises only secreted proteins. Both ADAM and ADAMTS proteins show a multi-domain structure and are mainly localised in the extracellular matrix (ECM) (Zhong and Khalil, 2019). Despite their structural similarities, each protein member possesses different variable domains which ensure both function and tissue specificity (Supplementary Figure 1). The domain organisation and function of each ADAM and ADAMTS protein has been extensively described elsewhere (Takeda, 2016). Briefly, ADAM proteins are responsible for shedding cell-surface protein ectodomains, such as the latent forms of growth factors, cytokines, receptors, and other molecules. Furthermore, ADAMs contribute to a wide array of biological processes, including cell adhesion, migration and signaling (Huovila et al., 2005; Seegar and Blacklow, 2019). From the twenty-one human ADAM members identified so far, only thirteen are proteolytically active (ADAM−8, −9, −10, −12, −15, −17, −19, −20, −21, −28, −30, −33, and −DEC1) whereas the other eight appear to be catalytically inactive (ADAM−2, −7, −11, −18, −22, −23, −29, and −32) (Edwards et al., 2009; Seegar and Blacklow, 2019). It has been shown that members of the latest group play important roles in development, and function as adhesion molecules rather than proteinases. However, the physiological function of the inactive ADAMs remains largely unknown.

Unlike ADAMs, all ADAMTS proteins are catalytically active and contain a varying number of C-terminal thrombospondin type-1 (TSP-1) motifs instead of the ADAM transmembrane and cytoplasmic domains (Apte, 2020). ADAMTSs participate in ECM maintenance, tissue morphogenesis and remodeling by cleaving a large number of matrix proteins (Kelwick et al., 2015; Apte, 2020). ADAMTS family consists of nineteen members that can be sub-classified according to their known substrates, namely aggrecanases or proteoglycanases (ADAMTS−1, −4, −5, −8, −9, −15 and −20), procollagen N-propeptidases (ADAMTS−2, −3 and −14), cartilage oligomeric matrix protein (also known as thrombospondin-5) cleaving proteinases (ADAMTS−7 and −12), von Willebrand factor (VWF) cleaving proteinase (ADAMTS-13) and a group of orphan enzymes (ADAMTS−6, −10, −16, −17, −18 and −19) (Kelwick et al., 2015; Apte, 2020).

Aberrant expression or dysregulation of ADAMs and ADAMTSs activity has been linked to the development of cancer (Sun et al., 2015; Jackson et al., 2017) and numerous inflammatory (Lambrecht et al., 2018; Mead and Apte, 2018), neurodegenerative (Duffy et al., 2009; Lemarchant et al., 2013) and cardiovascular (Zhong and Khalil, 2019; Santamaria and de Groot, 2020) diseases to name but a few. Hence, ADAM and ADAMTS proteins represent important drug targets for the prevention and treatment of several human diseases.

Inhibition of ADAM and ADAMTS metalloproteinases have been attempted using various small molecules. The majority of these molecules bear either the hydroxamate, carboxylate, thiolate, tartrate, phosphinate, thiadiazole, hydroxyquinoline or imida-zolidine-2,4-diones groups, which are capable of competitively binding the zinc ion present in the catalytic site of the metalloproteinase (Moss et al., 2001; Georgiadis and Yiotakis, 2008; Yiotakis and Dive, 2009). In addition, inhibitory molecules lacking a zinc-binding moiety have also been reported (Gilbert et al., 2011). Despite the different approaches attempted, these conventional small molecule-based inhibitors have mostly had limited success in the clinic (Georgiadis and Yiotakis, 2008; Moss and Minond, 2017). Failures have often been attributed to off-target effects due to structural similarities among the active sites of the different metalloproteinases and the consequent toxicities associated (Supplementary Figure 2 **)** (Georgiadis and Yiotakis, 2008; Tortorella et al., 2009; Raeeszadeh-Sarmazdeh et al., 2020). As a result, there is a great interest in developing novel ADAM and ADAMTS inhibitors that can selectively target a single member of each family. Efforts to generate more effective therapies have led to the development of protein-based inhibitors such as monoclonal antibodies and tissue inhibitors of metalloproteinases (TIMPs) which are currently being tested in advanced clinical trials (Santamaria and de Groot, 2019; Raeeszadeh-Sarmazdeh et al., 2020). Unlike small molecule-based inhibitors, protein-based therapeutics offer a higher selectivity due to a larger surface of interaction and therefore, reduced toxicity. Indeed, most protein-based inhibitors do not bind the active site of the ADAM and ADAMTS enzymes but recognise surface-exposed loops that are poorly conserved between closely related family members. Inhibition appears to occur through a variety of mechanisms including i) binding at or near the active site to block substrate access (direct manner) or ii) binding to regions that are allosterically linked to the active site region (indirect manner) (Wu et al., 2007; Raeeszadeh-Sarmazdeh et al., 2020). A major drawback of protein-based therapeutics compared to small molecule inhibitors is that they are not orally available and therefore need to be injected either subcutaneously or intravenously.

While most of these small-molecules and protein-based inhibitors have been thoroughly described elsewhere (Moss et al., 2001; Georgiadis and Yiotakis, 2008; Yiotakis and Dive, 2009; Murumkar et al., 2010; Gilbert et al., 2011; El Bakali et al., 2014; Santamaria et al., 2017; Malemud, 2019) this mini review focuses exclusively on peptide-based inhibitors, an alternative and emerging type of ADAMs and ADAMTSs metalloproteinase inhibitors. Similar to the protein-based inhibitors, peptides inhibitors are capable of binding the target with a surface of interaction large enough to obtain high efficiency and selectivity (Pelay-Gimeno et al., 2015; Atangcho et al., 2019). Like small molecules, peptide-based inhibitors can be synthesised chemically, possess ease of modification, low toxicity, and reduced antigenicity. Their modular structure and the commercial availability of hundreds of amino acid building blocks simplifies the rapid development of peptides with tailored properties (Rastogi et al., 2018; Muttenthaler et al., 2021). Here, we will mention examples of both linear and cyclic peptide-based inhibitors and the different approaches undertaken for their development will be described.

## PEPTIDE-BASED INHIBITORS OF ADAMs

**FIGURE 1:**
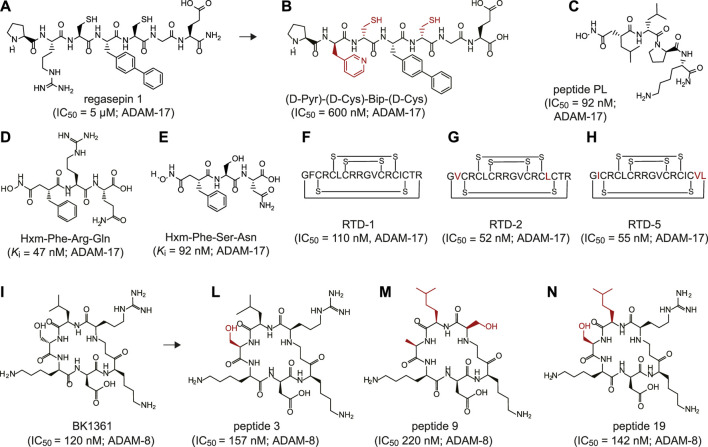
Peptide inhibitors of ADAM proteins. **(A)** Chemical structure of regasepin 1 linear peptide (PRC(Bip)CGE); **(B)** Chemical structure of regasepin 1-derived linear peptide (D-Pyr)-(D-Cys)-Bip-(D-Cys); **(C)** Chemical structure of peptide PL (Hisb-LPK-NH2); **(D)** Chemical structure of linear peptide Hxm-FRQ; **(E)** Chemical structure of linear peptide Hxm-FSN; **(F)** Schematically depicted structure of polycyclic octadecapeptide rhesus θ-defensin-1 (RTD-1) peptide; **(G)** Schematically depicted structure of polycyclic octadecapeptide rhesus θ-defensin-2 (RTD-2) peptide; **(H)** Schematically depicted structure of polycyclic octadecapeptide rhesus θ-defensin-5 (RTD-5) peptide; **(I)** Chemical structure of cyclic peptide BK1361 (RLsKDK); **(L)** Chemical structure of cyclic peptide 3 (RLhS_β_KDK); **(M)** Chemical structure of cyclic peptide 9 (RL*A_β_KDK); **(N)** Chemical structure of cyclic peptide 19 (RL*hS_β_KDK). Indicated half maximal inhibitory concentration (IC_50_) and inhibition constant (*K*i) values were reported as published. The targeted ADAM protein is reported near the IC_50_ or *K*_i_ value. Legend: Bip = biphenylalanine, Pyr = pyridylalanine, Hisb = (R)-2-isobutylsuccin hydroxamate moiety, Hmx = hydroxamate moiety, s = D-serine; hS_β_ = β-homoserine, A_β_ = β-alanine, L* = homoleucine. The side chains that differentiate from the parental peptide are shown in red.

To date, peptide-based inhibitors have been successfully developed against only two members of the ADAM family: ADAM-8 and ADAM-17 (Figure 1 and Supplementary Table 1). The latter one, also known as tumor necrosis factor-α converting enzyme (TACE), is involved in shedding the proinflammatory cytokine tumor necrosis factor-a (TNF-α) at the cell surface (Zunke and Rose-John, 2017). Altered activity of ADAM-17 has been associated with the onset of numerous inflammatory diseases, such as cardiac hypertrophy, arthritis, Chron’s disease and cancer (Feldmann and Maini, 2008; Moss and Minond, 2017; Lambrecht et al., 2018). The first peptide-based inhibitors targeting ADAM-17 were identified using synthetic combinatorial libraries of seven amino acids-long peptidomimetics (Hu et al., 2005a; Hu et al., 2005b). Libraries were designed to mimic the sequence of the cleavage sites in denatured collagen type II and include zinc-ion chelating side-groups. The screening revealed two low micromolar inhibitors, named regasepin 1 (Figure 1A) and regasepin 2, that inhibit related metalloproteinases MMP-8 and MMP-9 with similar potency (Supplementary Table 1; (Hu et al., 2005b; Dillen et al., 2006)). Further structure-based optimisation of regasepin 1 led to the generation of a small peptide repertoire bearing different D-form amino acids in place of Val and Lys in position P1′ at P2′, respectively. The best selected peptide, named (D-Pyr)-(D-Cys)-Bip-(D-Cys) (Figure 1B), showed an 8-fold higher potency (IC_50_ = 600 nM) than regasepin 1, 14-fold better selectivity against MMP-9 and 46-fold against MMP-3. However, no specificity for MMP-8 has been shown yet (Supplementary Table 1; (Qiu et al., 2012)). Similarly, Geurink and colleagues applied a synthetic combinatorial library of ninety-six enantiopure peptidomimetics bearing a zinc binding hydroxamate group at the N-terminal and identified eight peptide variants capable of inhibiting ADAM-17 with potencies in the sub-nanomolar to low micromolar range (Geurink et al., 2008). One peptide, named PL (Figure 1C), revealed an IC_50_ of 92 nM against ADAM-17 and showed 40-fold better selectivity against MMP-9 but limited selectivity for MMP-12 (Supplementary Table 1). By using a virtual combinatorial library of peptides derived from the TNF protease inhibitor 2 (TAPI-2), a broad-spectrum peptide inhibitor of ADAM-17 bearing a hydroxamate group, Wang and colleagues identified two linear peptides, named Hxm-Phe-Arg-Gln (Figure 1D) and Hxm-Phe-Ser-Asn (Figure 1E) that exhibited high potency toward ADAM-17 (*K*_i_ = 47 and 92 nM, respectively) and moderate selectivity toward ADAM-10 (5-fold and 7-fold, respectively; Supplementary Table 1; (Wang et al., 2016)). Furthermore, Schaal and colleagues discovered novel peptide-based inhibitors of ADAMs by screening θ-defensins, a family of eighteen-amino acid macrocyclic peptides expressed exclusively in granulocytes and selected epithelia of Old World monkeys (Schaal et al., 2017)). The octadecapeptide rhesus θ-defensin-1 (RTD-1) includes six disulfide-linked cysteines (Figure 1F) and inhibited ADAM-17 and ADAM-10 with an IC_50_ of 110 and 450 nM, respectively (Supplementary Table 1). Notably, RTD-1 showed at least 4-fold better selectivity toward a panel of related MMPs (IC_50_ = 2–20 μM; Supplementary Table 1). When tested *in vivo* in a rodent model of rheumatoid arthritis, RTD-1 rapidly suppressed joint disease progression, restored limb mobility, and preserved normal joint architecture (Schaal et al., 2017). Further characterisation of five RTD isoforms (RTDs 1–5) revealed the presence of two macrocycles, RTD-2 (Figure 1G) and RTD-5 (Figure 1H), that inhibited ADAM-17 with IC_50_ values of 52 and 55 nM, respectively (Supplementary Table 1; (Schaal et al., 2018)).

Another widely investigated sheddase is ADAM-8, a proteolytically active member of the ADAM protease family involved in numerous inflammatory processes (Schlomann et al., 2000) and neoplasia (Fritzsche et al., 2006; Romagnoli et al., 2014). By applying structural modeling and peptidomimetic approaches, Schlomann and colleagues generated a series of six amino acids cyclic peptides mimicking the RLSKDK motif of the mouse ADAM-8 (Schlomann et al., 2015). To enhance its potency and further increase its stability *in vivo*, the peptide sequence was constrained through cyclisation and modified with D-form amino acids in place of Arg, Leu, or Ser. The most potent cyclic peptide sequence contains a D-Ser (“s”) (RLsKDK) (Figure 1I). The RLsKDK, also named BK-1361, inhibited ADAM-8 with IC_50_ of 182 nM. Importantly, BK-1361 showed more than 100-fold better selectivity toward other related metalloproteinases such as ADAM−9, −10, −12, −17 and MMP-2, −9, −14 (Supplementary Table 1). Notably, when tested in a mouse model of pancreatic ductal adenocarcinoma (PDAC), BK-1361 led to reduction of tumor load, infiltration and metastasis. Thus, further supporting the important role of ADAM-8 in PDAC development (Schlomann et al., 2015). Additional structure-activity relationship studies on BK-1361 enabled the generation of eighteen structural analogue peptidomimetics (Yim et al., 2016). Among all tested cyclic peptides, peptides 3 (Figure 1L), 9 (Figure 1M) and 19 (Figure 1N) showed comparable or slightly higher inhibitory potency than the parental BK-1361 (Supplementary Table 1) (Yim et al., 2016).

## PEPTIDE-BASED INHIBITORS OF ADAMTSs

The physiological function of ADAMTSs and their role in numerous pathologies have been described only recently (Zhong and Khalil, 2019; Apte, 2020). The first member of this family, ADAMTS-1, was characterised for the first time in 1997 (Kuno et al., 1997). Since then, few examples of peptide-based inhibitors against ADAMTSs have been reported (Tortorella et al., 2000; Hills et al., 2007; Di Stasio et al., 2008; Moriki et al., 2010; Pillai et al., 2016; Zhang et al., 2018).

**FIGURE 2:**
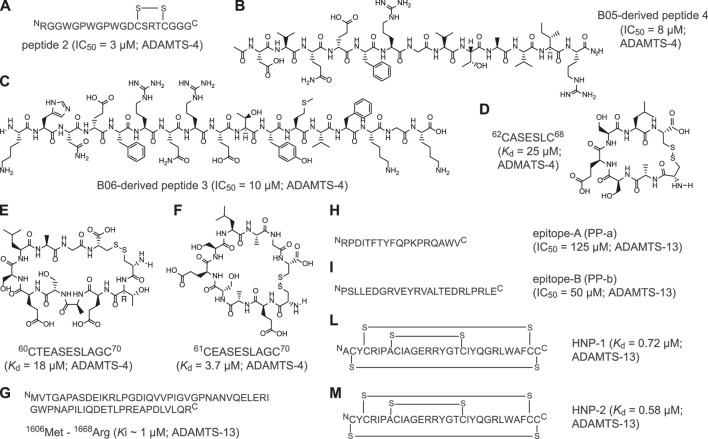
Peptide inhibitors of ADAMTS proteins. **(A)** Aminoacidic sequence of linear peptide 2 derived from the C-terminus TSP-1 motif of aggrecanase-1; **(B)** Chemical structure of B05-derived linear peptide 4 (KHN(e)FRQRETYMVFKGK); **(C)** Chemical structure of B06-derived linear peptide 3 (DVQ(e)FRGVTAVIR); **(D)** Chemical structure of cyclic peptide ^62^CASESLC^68^; **(E)** Chemical structure of cyclic peptide ^60^CTEASESLAGC^70^; **(F)** Chemical structure of cyclic peptide ^61^CEASESLAGC^70^; **(G)** Aminoacidic sequence of linear peptide VWF-73 peptide (Met^1606^-Arg^166^) derived from C-terminal region of the multimeric von Willebrand factor (VWF); **(H)** Aminoacidic sequence of linear peptide epitope-A (PP-a); **(I)** Aminoacidic sequence of linear peptide epitope-B (PP-b); **(L)** Aminoacidic sequence of polycyclic human neutrophil peptide 1 (HNP-1); **(M)** Aminoacidic sequence of polycyclic human neutrophil peptide (HNP-2). Indicated half maximal inhibitory concentration (IC_50_), inhibition constant (*K*_i_) and dissociation constant (*K*_d_) values were reported as published. The targeted ADAMTS protein is reported near the IC_50_, *K*_i,_ or *K*_d_ value.

Recently, major efforts have been devoted to developing peptide-based inhibitors against two members of the ADAMTS family: ADAMTS-4 and ADAMTS-13 (Figure 2 and Supplementary Table 2). ADAMTS-4 cleaves proteoglycans such as aggrecan and versican, which play a structural role in many tissues (Fosang and Little, 2008). In fact, degradation of aggrecan is a clinical hallmark of degenerative joint disorders such as osteoarthritis Zhang et al. (2013), Yang et al. (2017) and rheumatoid arthritis (Mead and Apte, 2018). The first peptide-based inhibitors targeting ADAMTS-4 were identified using linear peptides derived from the TSP-1 motif located at the C-terminus of the aggrecanase-1, an enzyme involved in cartilage degradation. The best selected peptide, peptide 2 (Figure 2A), showed an IC_50_ of 3 µM against ADAMTS-4 (Supplementary Table 2; (Tortorella et al., 2000)). With the aim of identifying the cleavage motif of ADAMTS-4, Hills and co-workers applied phage display of random thirteen-amino acid linear peptide libraries to isolate seven-amino acid cleaved peptides with a wide range of potencies (Hills et al., 2007). Two linear peptides, B05 and B06, inhibited ADAMTS-4 with potencies in the micromolar range (IC_50_ = 35 μM) and exhibited good selectivity toward the homologue ADAMTS-5 (Supplementary Table 2; Hills et al., 2007). Further studies revealed the importance of Glu in position P1 for substrate recognition and led to the development of novel synthetic peptides with modified stereochemistry of P1 and P1'. These two selected peptides inhibited ADAMTS-4 with IC_50_ values of 8 μM (peptide 4, Figure 2B) and 10 μM (peptide 3, Figure 2C) (Hills et al., 2007). Recently, Zhang and colleagues used computational modeling to develop peptide-based inhibitors from a loop of the N-terminal domain of TIMP3, a protein inhibitor of ADAMTS-4 (Zhang et al., 2018). Further peptide cyclisation diminished flexibility and enabled the generation of constrained molecules with reduced entropic penalty and improved binding affinities. Cyclised peptides ^62^CASESLC^68^ (Figure 2D), ^61^CEASESLAGC^70^ (Figure 2F) and ^60^CTEASESLAGC^70^ (Figure 2E) showed bindings constants in the micromolar range (*K*_d_ = 25 μM, *K*_d_ = 3.7 μM and *K*_d_ = 18 μM, respectively) and three- to 9-fold increased affinity over the linear peptides (Supplementary Table 2).

In addition to ADAMTS-4, peptide-based inhibitors against ADAMTS-13 have been also developed. ADAMTS-13 is a metalloproteinase which cleaves the von Willebrand factor (VWF), a blood glycoprotein involved in haemostasis (Zheng, 2013). The levels of ADAMTS-13 correlate with ischaemic stroke risk, thrombotic thrombocytopenic purpura and microvascular thrombosis (South and Lane, 2018; Santamaria and de Groot, 2020). The first peptide-based inhibitor of ADAMTS-13 was identified by analysing the C-terminal region of the multimeric VWF factor, the VWF-73 peptide (Glu^1660^—Arg^1668^; Figure 2G) (Di Stasio et al., 2008). By elucidating the interaction of linear VWF-73 peptide with ADAMTS-13, Di Stasio and colleagues determined that inhibition occurs with a *K*_i_ value of 1 μM (Supplementary Table 2). Furthermore, Moriki and colleagues applied phage display technology to identify two novel ADAMTS-13-derived peptide epitopes capable of binding VWF factor. Selected synthetic linear peptides PP-a and PP-b (Figures 2H,I) exhibited *K*_d_ values of 4.1 and 0.3 µM, respectively, and inhibited ADAMTS-13 with IC_50_ values of 125 μM (PP-a) and 50 μM (PP-b) (Supplementary Table 2) (Moriki et al., 2010). Finally, Pillai and co-workers showed that polycyclic human neutrophil peptides (HNP) inhibit the proteolytic cleavage of peptide VWF-73 and multimeric von Willebrand factor in a concentration-dependent manner. HNP-1 and -2 (Figures 2L,M) showed inhibitory concentrations in the low micromolar range and binding constants in the sub-micromolar range (Supplementary Table 2) (Pillai et al., 2016).

## Conclusions and Perspectives

ADAM and ADAMTS metalloproteinases play a significant yet complex role in several types of cancer, as well as in diverse inflammatory, neurodegenerative and cardiovascular diseases. Thus, a plethora of small chemical molecules and a few large proteins, such as monoclonal antibodies, have been developed to inhibit ADAM and ADAMTS metalloproteinases. While small chemical molecules often lack specificity and turn to be toxic, therapeutic proteins require high manufacturing costs and subcutaneous or intravenous administration. In this sense, peptide-based drugs offer a good alternative strategy with a surface of interaction large enough to obtain both high potency and selectivity, and yet small enough to diffuse into tissues. Other distinctive properties of peptides include chemical synthesis, ease of modification, low toxicity and reduced antigenicity. However, despite these favourable traits, peptides often have a relatively short circulating half-life and exhibit poor membrane permeability, which limit their broad applicability. While their systemic half-life can be prolonged by chemical conjugation to synthetic and natural polymers, or through non-covalent binding to endogenous proteins, such as serum albumin (Zorzi et al., 2019), reaching intracellular targets, on the other hand, is still a daunting task for peptide-based drugs. Although recent developments in chemical cyclization, methylation and the use of non-proteinogenic amino acids have led to promising results to overcome this problem, more accessible targets would also help to bypass the delivery strategies challenges (Cunningham et al., 2017; Lenci and Trabocchi, 2020). In this regard, ADAM and ADAMTS proteins have a peripheral extracellular localisation, which makes them ideal targets of peptide-based drugs. Moreover, the existence of multiple ADAM and ADAMTS homologues leverage the better selectivity of peptides (driven by their larger surface area and chiral complexity) over small-molecule drugs. The majority of linear and cyclic peptide inhibitors described in this mini review were developed using traditional medicinal chemistry approaches and structure–activity relationship studies on natural substrates and/or endogenous inhibitors. However, the specificity of some of the peptide inhibitors described here have not been fully investigated and none of them have reached a pre-clinical stage yet. Nevertheless, their development demonstrates that peptides represent valid molecular modalities for blocking the activity of ADAM and ADAMTS proteins. Indeed, the advent of novel DNA-encoded chemical libraries (Neri and Lerner, 2018) and superior peptide display technologies (Linciano et al., 2019; Sohrabi et al., 2020; Peacock and Suga, 2021) will enable the high-throughput screening of large combinatorial libraries, facilitating the discovery of novel potent and selective compounds with improved properties on short timescales (Sohrabi et al., 2020). Integration of these powerful combinatorial approaches with better automation, innovative chemical modification strategies and emerging computational methods will contribute to the development of better peptide-based inhibitors against ADAM and ADAMTS proteins, which have the potential to be used in the clinic in the near future.
