# Supplementary material for: Peptide-Based Inhibitors of ADAM and ADAMTS Metalloproteinases
Source: Front Mol Biosci. 2021 Jul 21;8:703715. doi: 10.3389/fmolb.2021.703715 (PMC8335159; doi:10.3389/fmolb.2021.703715)
Supplement: Supplementary file 1 [file DataSheet1.pdf]

Supplementary Material

1. Supplementary Tables

Target	Peptide name	Potency	Specificity	Reference
ADAM-17	regasepin 1	IC_{50} = 5 µM†	20-folds MMP-1 and MMP-13<br><br>IC_{50} = 3 µM MMP-8 and MMP-9	(Hu et al., 2005)
	(D-Pyr)-(D-Cys)-Bip-(D-Cys)	IC_{50} = 600 nM†	14-folds MMP-9, 46-folds MMP-3,<br><br>IC_{50} = 730 nM MMP-8	(Qiu et al., 2012)
	peptide PL	IC_{50} = 92.1 ± 28 nM	40-folds MMP-9, 1.5-fold MMP-12	(Geurink et al., 2008)
	Hxm-Phe-Ser-Asn	K_i = 92 ± 14 nM	7-fold ADAM-10	(Wang et al., 2016)
	Hxm-Phe-Arg-Gln	K_i = 47 ± 6 nM	5-fold ADAM-10	
	RTD-1	IC_{50} = 110 ± 40 nM	>50-folds MMP-1, MMP-2, MMP-3, MMP-8, MMP-9, MMP-13 and MMP-14<br><br>IC_{50} = 0.45 ± 0.2 µM ADAM-10	(Schaal et al., 2017)

	RTD-2	IC_{50} = 52 ± 3 nM	n.a.	(Schaal et al., 2018)
	RTD-5	IC_{50} = 55 ± 7 nM	n.a.	
ADAM-8	BK-1361	IC_{50} = 120 ± 19 nM<br>IC_{50} = 182 nM	> 100-folds ADAM-9, ADAM-10, ADAM-12, ADAM-17, MMP-2, MMP-9 and MMP-14	(Schlomann et al., 2015)<br>(Yim et al., 2016)
	peptide 3	IC_{50} = 157 ± 32 nM	n.a.	(Yim et al., 2016)
	peptide 9	IC_{50} = 220 ± 28 nM	n.a.	
	peptide 19	IC_{50} = 142 ± 21 nM	n.a.	

Table 1. Peptide inhibitors of ADAM targets. ADAM target, peptide name, amino acid sequences, potency and specificity are reported. Indicated half maximal inhibitory concentration (IC_{50}) and inhibition constant (K_i) values were reported as published. Legend: Bip = biphenylalanine, Pyr = pyridylalanine, Hisb = (R)-2-isobutylsuccin hydroxamate moiety, Hmx = hydroxamate moiety, s = D-serine; hS_β = β-homoserine, A_β = β-alanine, L* = homoleucine. Note: † = standard deviation not reported, n.a. = not available.

Target	Peptide inhibitor	Potency	Specificity	Reference
ADAMTS-4	peptide 2	$IC_{50} = 3\ \mu M^{\dagger}$	n.a.	(Tortorella et al., 2000)
	peptides 3 based on peptide B06	$IC_{50} = 10\ \mu M^{\dagger}$	n.a.	(Hills et al., 2007)
	peptides 4 based on peptide B05	$IC_{50} = 8\ \mu M^{\dagger}$	n.a.	
	^{62}CASESLC^{68}	$K_d = 25 \pm 4\ \mu M$	n.a.	(Zhang et al., 2018)
	^{61}CEASESLAGC^{70}	$K_d = 3.7 \pm 0.5\ \mu M$	n.a.	
	^{60}CTEASESLAGC^{70}	$K_d = 18 \pm 4\ \mu M$	n.a.	
ADAMTS-13	Met^{1606}-Arg^{1668}	$K_i \sim 1\ \mu M^{\dagger}$	n.a.	(Di Stasio et al., 2008)
	epitope-A (PP-a)	$IC_{50} = 125\ \mu M^{\dagger}$	n.a.	(Moriki et al., 2010)
	epitope-B (PP-b)	$IC_{50} = 50\ \mu M^{\dagger}$	n.a.	
	HNP-1	$K_d = 0.72\ \mu M^{\dagger}$	n.a.	(Pillai et al., 2016)
	HNP-2	$K_d = 0.58\ \mu M^{\dagger}$	n.a.	

Table 2. Peptide inhibitors of ADAMTS targets. ADAMTS target, peptide name, amino acid sequences, potency and specificity are reported. Indicated half maximal inhibitory concentration (IC_{50}), inhibition constant (K_i) and dissociation constant (K_d) values were reported as published. Note: † = standard deviation not reported, n.a. = not available.

2. Supplementary Figures

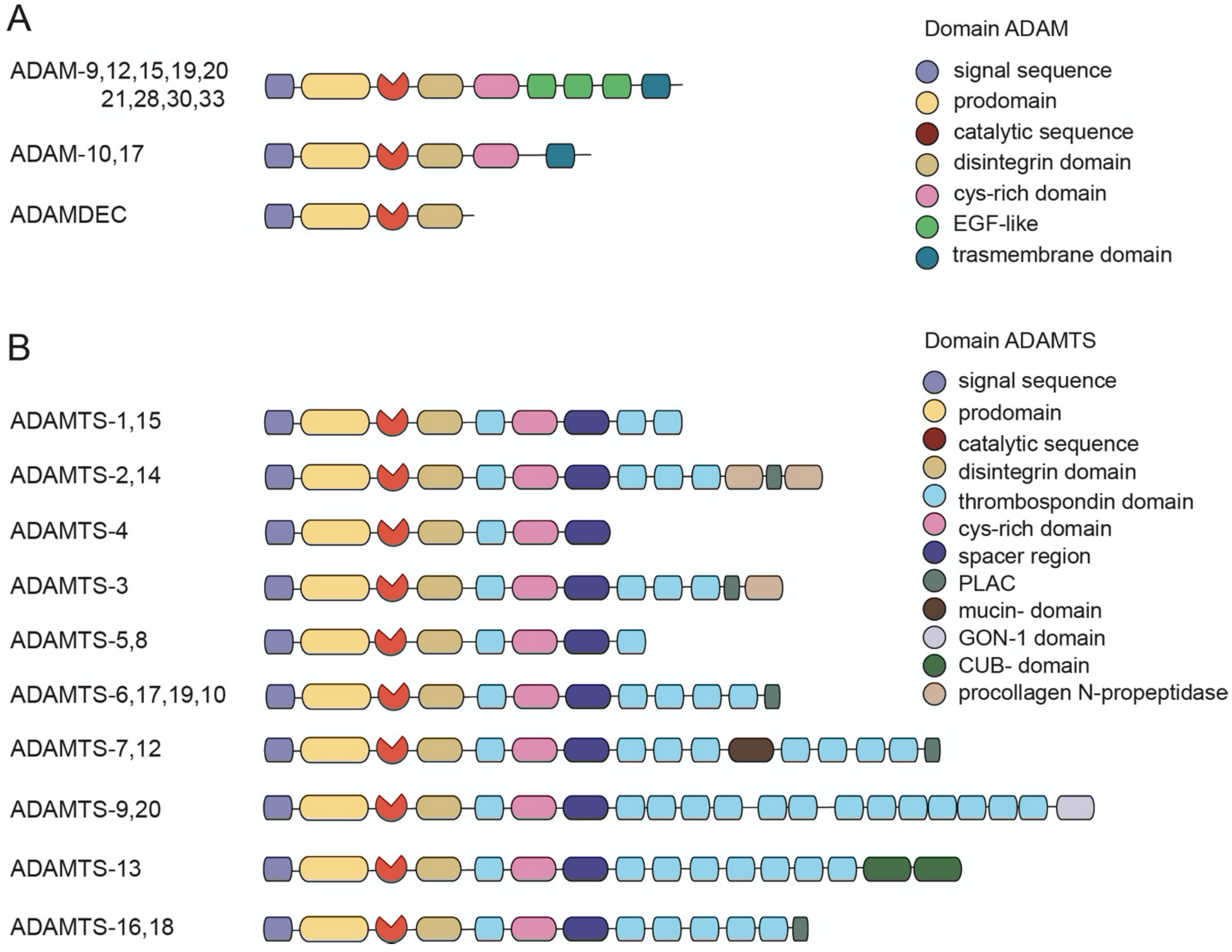

Supplementary Figure 1. Domain diagram of the catalytically active members of human ADAM and ADAMTS families. A) ADAM proteins generally include, from N- to C-terminus, a specific signal secretion sequence (purple), a prodomain (yellow) containing highly conserved cysteine that block the proteolytic activity of the metalloproteinase catalytic domain (brown) by preferentially binding to the zinc atom within the active center, a disintegrin-like domain (light brown), a cysteine-rich domain (pink) containing a hypervariable region (HVR), an epidermal growth factor domain-like (EGF-like) repeat domain (light green), a short connecting linker, a hydrophobic transmembrane segment (dark green) and a cytoplasmic tail. **B**) ADAMTS members commonly possess, from the N- to C-terminus, a signal secretion sequence (purple), a prodomain (yellow), a catalytic domain (brown), a disintegrin-like domain (light brown), a central thrombospondin motif (light blue), a cysteine-rich domain (pink) and a spacer region (dark purple). The major difference between ADAMTS members

lies in the ancillary domains including one or more thrombospondin sequences and one or more specialized domains such as protease and lacunin domain (PLAC), mucin domain, GON-1 domain, CUB domain and procollagen N-propeptidase.

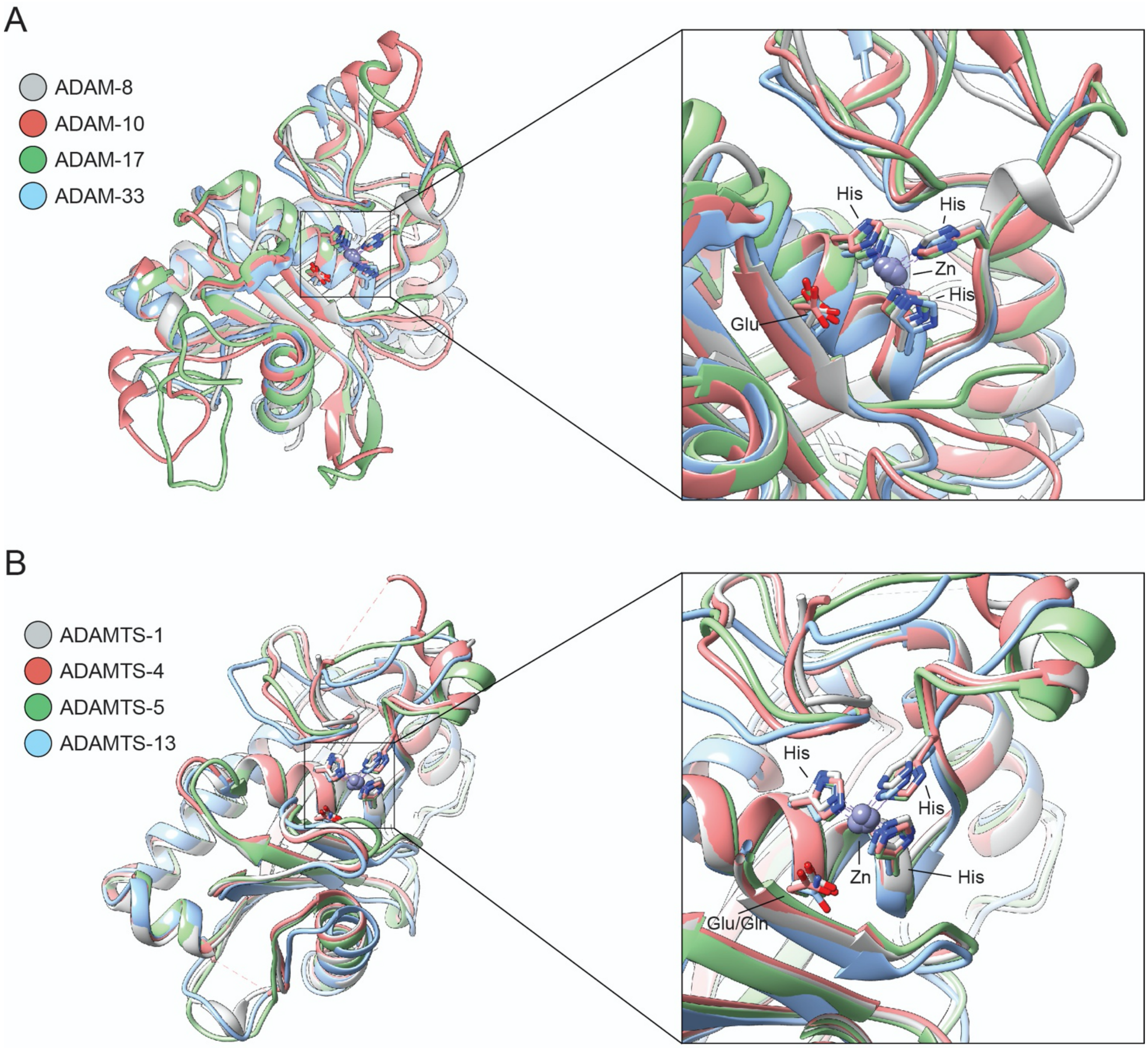

Supplementary Figure 2. Structural comparison of the tridimensional structures of the catalytic domains of ADAM and ADAMTS proteins. **A**) Superimposed X-ray crystal structures of ADAM-8 (gray; PDB identification code 4DD4), ADAM-10 (red; PDB identification code 6BE6), ADAM-17 (green; PDB identification code 2DDF) and ADAM-33 (light blue; PDB identification code 1R55); **B**) Superimposed X-ray crystal structures of ADAMTS-1 (gray; PDB identification code 2V4B), ADAMTS-4 (red; PDB identification code 3B2Z), ADAMTS-5 (green; PDB identification code 3B8Z) and ADAMTS-13 (light blue; PDB identification code 6QIG). The zoomed-in view of the active sites of both ADAM and ADAMTS structures highlights the similarities of the catalytic pockets including a glutamate (Glu) or glutamine (Gln) residue and three conserved histidine (His) residues that coordinate the zinc ion (light blue sphere).

References:

Di Stasio, E., Lancellotti, S., Peyvandi, F., Palla, R., Mannucci, P. M., and De Cristofaro, R. (2008). Mechanistic studies on ADAMTS13 catalysis. *Biophys. J.* 95, 2450–2461. doi:10.1529/biophysj.108.131532.

Geurink, P., Klein, T., Leeuwenburgh, M., Van Der Marel, G., Kauffman, H., Bischoff, R., et al. (2008). A peptide hydroxamate library for enrichment of metalloproteinases: Towards an affinity-based metalloproteinase profiling protocol. *Org. Biomol. Chem.* 6, 1244–1250. doi:10.1039/b718352f.

Hills, R., Mazzarella, R., Fok, K., Liu, M., Nemirovskiy, O., Leone, J., et al. (2007). Identification of an ADAMTS-4 cleavage motif using phage display leads to the development of fluorogenic peptide substrates and reveals matrilin-3 as a novel substrate. *J. Biol. Chem.* 282, 11101–11109. doi:10.1074/jbc.M611588200.

Hu, J., Van Den Steen, P. E., Dillen, C., and Opdenakker, G. (2005). Targeting neutrophil collagenase/matrix metalloproteinase-8 and gelatinase B/matrix metalloproteinase-9 with a peptidomimetic inhibitor protects against endotoxin shock. *Biochem. Pharmacol.* 70, 535–544. doi:10.1016/j.bcp.2005.04.047.

Moriki, T., Maruyama, I. N., Igari, A., Ikeda, Y., and Murata, M. (2010). Identification of ADAMTS13 peptide sequences binding to von Willebrand factor. *Biochem. Biophys. Res. Commun.* 391, 783–788. doi:10.1016/j.bbrc.2009.11.138.

Qiu, Z., Yan, M., Li, Q., Liu, D., Van den Steen, P. E., Wang, M., et al. (2012). Definition of peptide inhibitors from a synthetic peptide library by targeting gelatinase B/matrix metalloproteinase-9 (MMP-9) and TNF-α converting enzyme (TACE/ADAM-17). *J. Enzyme Inhib. Med. Chem.* 27, 533–540. doi:10.3109/14756366.2011.599323.

Schaal, J. B., Maretzky, T., Tran, D. Q., Tran, P. A., Tongaonkar, P., Blobel, C. P., et al. (2018). Macrocyclic θ-defensins suppress tumor necrosis factor-α (TNF-α) shedding by inhibition of TNF-α-converting enzyme. *J. Biol. Chem.* 293, 2725–2734. doi:10.1074/jbc.RA117.000793.

Schaal, J. B., Tran, D. Q., Subramanian, A., Patel, R., Laragione, T., Roberts, K. D., et al. (2017). Suppression and resolution of autoimmune arthritis by rhesus θ-defensin-1, an immunomodulatory macrocyclic peptide. *PLoS One* 12, e0187868. doi:10.1371/journal.pone.0187868.

Schlomann, U., Koller, G., Conrad, C., Ferdous, T., Golfi, P., Garcia, A. M., et al. (2015). ADAM8 as a drug target in pancreatic cancer. *Nat. Commun.* 6, 1–16. doi:10.1038/ncomms7175.

Tortorella, M., Pratta, M., Liu, R. Q., Abbaszade, I., Ross, H., Burn, T., et al. (2000). The thrombospondin motif of Aggrecanase-1 (ADAMTS-4) is critical for aggrecan substrate recognition and cleavage. *J. Biol. Chem.* 275, 25791–25797. doi:10.1074/jbc.M001065200.

Wang, Z., Wang, L., Fan, R., Zhou, J., and Zhong, J. (2016). Molecular design and structural optimization of potent peptide hydroxamate inhibitors to selectively target human ADAM metallopeptidase domain 17. *Comput. Biol. Chem.* 61, 15–22. doi:10.1016/j.compbiolchem.2015.12.003.

Yim, V., Noisier, A. F. M., Hung, K. yuan, Bartsch, J. W., Schlomann, U., and Brimble, M. A. (2016). Synthesis and biological evaluation of analogues of the potent ADAM8 inhibitor cyclo(RLsKDK) for the treatment of inflammatory diseases and cancer metastasis. *Bioorganic Med. Chem.* 24, 4032–4037. doi:10.1016/j.bmc.2016.06.042.

Zhang, W., Zhong, B., Zhang, C., Wang, Y., Guo, S., Luo, C., et al. (2018). Structural modeling of osteoarthritis ADAMTS4 complex with its cognate inhibitory protein TIMP3 and rational derivation of cyclic peptide inhibitors from the complex interface to target ADAMTS4. *Bioorg. Chem.* 76, 13–22. doi:10.1016/j.bioorg.2017.10.017.
